# Factors associated with the job satisfaction of certified nurses and nurse specialists in cancer care in Japan: Analysis based on the Basic Plan to Promote Cancer Control Programs

**DOI:** 10.1371/journal.pone.0232336

**Published:** 2020-05-18

**Authors:** Masaki Kitajima, Chiharu Miyata, Keiko Tamura, Ayae Kinoshita, Hidenori Arai

**Affiliations:** 1 Human Health Sciences, Graduate School of Medicine, Kyoto University, Kyoto, Japan; 2 Nursing, Mie University Graduate School of Medicine, Mie, Japan; 3 National Center for Geriatrics and Gerontology, Obu, Aichi, Japan; Institute of Mental Health, SINGAPORE

## Abstract

**Background:**

As the Japanese population ages, the number of cancer patients will likely increase. Therefore, qualified cancer health care providers should be recruited and retained. Nurse job satisfaction is influenced by numerous factors and may affect staff turnover and patient outcomes.

**Objectives:**

To evaluate the job satisfaction of certified nurses and nurse specialists in Japanese cancer care and elucidate factors associated with job satisfaction.

**Methods:**

Participants in this cross-sectional study comprised 200 certified nurse specialists and 1,472 certified nurses working in Japanese cancer care. A chi-square test and logistic regression analysis were conducted to identify job satisfaction factors.

**Results:**

Job satisfaction was present in 38.45% and 49.00% of certified nurses and nurse specialists, respectively. Certified nurses associated job satisfaction with cross-departmental activities (OR 2.24, *p*<0.001), positive evaluation from senior stuff (OR 4.58, *p*<0.001), appropriate staff allocation (OR 1.75, *p*<0.001), more than five years certified nurse experience (OR 1.91, *p*<0.001), and positive evaluation of the development of certified nurses (OR 2.13, *p*<0.01) and nurse specialists (OR 1.37, *p*<0.05). Low job satisfaction was associated with working on a ward (OR 0.51, *p*<0.001) and a capacity of more than 200 beds (OR 0.33, *p =* 0.00). Certified nurse specialists associated job satisfaction with palliative care team participation (OR 2.64, *p*<0.05), cross–sectional activities (OR 7.06, *p*<0.01), positive evaluation from senior stuff (OR 13.15, *p*<0.001), presence of certified nurses in radiation therapy (OR 2.91, *p*<0.05), positive certified nurse specialist development evaluation (OR 7.35, *p*<0.001), medical service fees (OR 3.78, *p*<0.01), and independent activities (OR 11.34, *p*<0.01).

**Conclusions:**

We identified factors related to activities, facilities, and the cancer care team associated with job satisfaction of certified nurses and nurse specialists in Japanese cancer care. Suggestions are provided to enhance job satisfaction through Japan’s Basic Plan to Promote Cancer Control, which may help hospital administrators retain nursing staff.

## Introduction

With the aging of the global population, the number of cancer patients and cancer-related deaths in Japan is expected to increase [1]. To provide high-quality medical services and optimally support cancer patients and their families, the Basic Plan to Promote Cancer Control Programs was adopted by the Japanese national health care system. It suggests a collaborative approach involving multiple healthcare professionals.

It is important to ensure a sufficient staff of specialized care providers including nursing professionals with qualifications awarded by the Japan Nursing Association [1]. A certified nurse specialist (CNS) is a nurse who participates in clinical practice, consultation, coordination of activities, ethical management, education, and research; has at least five years of nursing experience; and has obtained certification after completing a master’s degree at a graduate school. Through their job activities, CNSs aim to improve the quality of medical care, propose policies, and work to maintain and improve patient health [2]. Therefore, they are often assigned to nursing administration offices and frequently involved in activities to improve the entire facility, such as staff education, research, and patient discharge.

A certified nurse (CN) is a nurse who participates in clinical practice, teaching, and consultation; has more than five years of nursing experience; and has obtained certification after graduating from a special vocational school approved by the Japan Nursing Association. CNs are generally assigned as staff nurses in a single department such as an outpatient clinic or a ward [3].

In accordance with the stipulations of the Basic Plan to Promote Cancer Control, CNSs and CNs are recruited to hospitals authorized to treat cancer to alleviate the burden on cancer patients and their families and improve the quality of recuperation. Furthermore, CNSs and CNs are assigned to the palliative care team and provide patient counseling. Because of the critical role of oncology CNSs and CNs in patient care, understanding the factors influencing their job satisfaction and performance has important implications for clinical practice. Since the Basic Plan to Promote Cancer Control stipulates the most important measures in the treatment and support of cancer patients, it is important to incorporate it in the evaluation of the job satisfaction of CNs and CNSs [4].

The job satisfaction of nurses has been addressed in other settings [5–10]. A recent integrative review showed that job satisfaction among nurses is a variable and complex phenomenon depending on numerous factors [7]. The factors varied between studies and included adequate staffing and equipment, job security and compensation, opportunities for professional development, supervisor support, autonomy, quality of workplace relationships, and the feeling that one’s job makes a difference.

Many studies evaluated the professional growth, role, support, and recognition of CNSs and CNs [11–21]. However, research on job satisfaction has only been conducted in the fields of psychiatric mental health nursing for CNSs and in dysphagia nursing for CNs [22–25]. CNSs are nursing specialists whose role requires substantial autonomy. Reportedly, the autonomous role of a caregiver improves the job satisfaction and performance of nurses [26–30]. Furthermore, the concept of a specialist implies autonomy including responsibility and expertise [31].

No study has evaluated job satisfaction among CNSs and CNs in the field of oncology in Japan. However, this information is crucial in the retention of nursing stuff and provision of optimal patient care, since job satisfaction presumably affects the expertise of CNSs and CNs and their ability to provide outstanding care. In addition, the specific job profiles and responsibilities of CNSs and CNSs may be associated with distinct job satisfaction factors. Identifying these factors may facilitate the implementation of specific measures to improve job satisfaction. Therefore, we assessed the job satisfaction of CNSs and CNs working in cancer care in Japan and identified factors enabling them to operate productively in their organizations.

## Methods

### Survey development and testing

Because CNSs and CNs have unique roles, existing scales could not be applied in this study. Previous reports were used to identify possible factors influencing job satisfaction for inclusion in the current investigation [22–25]. Furthermore, the Basic Plan to Promote Cancer Control was utilized to select questions pertaining to personnel deployment requirements for CNSs and CNs, facility types, facility requirements, medical care remuneration, and palliative care team placement requirements.

Items were divided into categories related to activities, facilities, and participation in the cancer care team. To examine the content validity of the questions, university faculty members well acquainted with the Basic Plan to Promote Cancer Control and activities of CNSs and CNs discussed and agreed on the included items. Next, a pretest was administered to four CNSs and four CNs, who were asked whether they faced any difficulties in answering the questions and understanding the terms used. The terminology was revised as necessary. Four researchers extracted the characteristic activities of the CNSs and CNs based on the Basic Plan to Promote Cancer Control. The extracted items and question items on job satisfaction were examined, and the relevance of each was investigated.

### Job satisfaction questionnaire: Design and content

The following are examples of questions asked: “Are cross-sectional activities conducted in the hospital?” “Is the personnel placement of the assigned department appropriate?” “Do you feel that you are well evaluated by your boss?” Furthermore, eleven questions concerned factors related to activities (including demographics), seven concerned factors related to facilities, and four concerned factors related to the cancer care team. Table 1 presents the questions. The study participants were asked to respond to the questionnaire survey on a simple three-point scale, which was used because of the difficulty in securing time during working hours in a busy clinical practice. The pretest was measured on a five-point scale, but it required extended time to answer and appeared to cause a significant clinical burden. A confirmatory factor analysis (CFA) was conducted to verify the factor structure of the questionnaire. A three-factor model was analyzed, including factors related to activities, facilities, and the cancer team. The following values were obtained for the model fit: Comparative fit index (CFI) 0.96, Tucker–Lewis index (TLI) 0.90, and root mean-square error of approximation (RMSEA) 0.04. Overall, the findings of the CFA indicated an acceptable fit of the three-factor model. Furthermore, the reliabilities of the three scales were estimated by calculating Cronbach’s alpha, which was 0.79, 0.81, and 0.72 for factors related to activities, facilities, and the cancer team, respectively.

**Table 1:** Contents of the questionnaire.

1. Factors related to activities (including demographics)
① Years of experience after acquiring nursing license (open-ended) ② Years of experience after acquiring CNS or CN qualifications (open-ended) ③ The presence or absence of other CNSs and CNs working in the same institution (open-ended)
④ Working system (multiple answers)^1^ (palliative care team, cancer consultation and support center, outpatient chemotherapy room, radiotherapy room, outpatient department, ward department)
⑤ Position (open-ended)
⑥ Lecturer for study group or training (frequently and regularly; yes, but not that frequently; no)
⑦ Experienced the launch of a department or patient group (yes, no)
⑧ Opportunity for cross-departmental activities^1^ (always; not always, but regularly; never)
⑨ Opportunity for exchange with CNSs and CNs in other institutions (regularly; yes, but not regularly; never)
⑩ Positive evaluation from senior staff (yes, sometimes, never) ⑪Appropriate staff allocation
2. Factors related to facilities
① Type of hospital (prefectural cancer center hospital, regional cooperation cancer center hospital, cancer center hospital designated by prefecture, community hospital, other)
② Bed capacity (0 to 199, 200 to 499, ≥ 500, other)
③ Publicize information about the existence of CNSs and CNs among community members and the public (yes, no)
④ Service system in the following four departments pertaining to cancer(chemotherapy room, palliative care team, cancer consultation and support center, radiotherapy room)
⑤ Additional medical service compensation for cancer (palliative care practice addition, cancer patient counseling charges, outpatient palliative care management charges, cancer pain palliation instruction charges)
⑥ Implementation of cancer care in the institution (based on the requirements of designated cancer centers and hospitals)
⑦ Positive evaluation of CNS and CN development in the institution (always, sometimes, never)
3. Factors related to the cancer care team
① Coordination among multiple healthcare professionals (always, sometimes, never)
② Independent activities^2^ (always, sometimes, never)
③ Availability of conferences (times per week) ④ Job type (pharmacist, medical social worker, nutritionist, physical therapist, occupational therapist, speech therapist, clinical psychologist)

Regarding department affiliations, if individuals were involved with two or more affiliations concurrently, they were instructed to choose multiple answers.

Refers to the ability to fulfill one’s role without interference from others.

In addition, we enquired about job satisfaction. We asked the question, “Are you satisfied with your job?” Originally, we used a three-level scale with the following response options: “satisfied,” “somewhat satisfied,” and “dissatisfied.” However, upon review, we combined the first two levels to yield a two-level scale: “satisfied” and “dissatisfied.”

### Procedure

This study was cross-sectional and involved the use of self-report questionnaires. A survey request, self-report questionnaire, document explaining the implications of the study, and return envelope were mailed to each participant. The recipients of the survey were asked to return the questionnaire to the researchers within two months. The survey period was between May and July 2014. In addition, a written notification was included informing the participants that their participation was voluntary. Precautions were taken to ensure that the department heads were unaware of whether the CNSs and CNs had mailed the completed questionnaires.

### Participants

In February 2014, 3,450 nurses were included on the list of registered CNSs and CNs on the official website of the Japan Nursing Association. Of these, 3,332 nurses (483 CNSs and 2,849 CNs) whose addresses could be confirmed were mailed the questionnaire. In total, 1,696 nurses working in cancer centers or hospitals involved in cancer care responded to the mailed questionnaire. The valid response rate was 98.60% (1,672 respondents).

### Ethical considerations

The study protocol was approved by the Institutional Ethics Committee of the Kyoto University Graduate School of Medicine and Faculty of Medicine Hospital with protocol number E2072. The purpose of the study, benefits of participation, confirmation of voluntary participation, and an assurance that all data obtained in the present study would be used only for scientific purposes and anonymously were communicated to all participants in written form. All participants gave their informed consent. To protect confidentiality, personal data were kept separately from the completed questionnaires, which were coded. The study adhered to the STROBE checklist.

### Data analysis

Descriptive statistics were computed for each questionnaire item. The three-point scale had the anchors *Carried out*, *Somewhat carried out*, and *Not carried out*. Responses were categorized in a binary format, in which *Carried out* and *Somewhat carried out* represented an affirmative *Yes* response (1), and *Not carried out* represented a *No* response (2). In addition, a “bed capacity” of 0–199 was assigned a score of 1, and a capacity ≥ 200 a score of 2. For the type of hospital, a cancer center was assigned a score of 1, and “other hospital” a score of 2. Conferences were scored 1 for “Held/Yes” or 2 for “Not held/No.”

A chi-square test was performed to evaluate the relationship between job satisfaction and other variables. Factors statistically significant in the univariate analysis were included in the subsequent multivariable analysis. To identify factors related to job satisfaction, a logistic regression analysis was conducted with satisfaction or dissatisfaction as the dependent variable, other factors as the explanatory variables, and years of experience after acquiring a nursing license as the control variable. The following explanatory variables were used in the case of categorical variables. A dummy variable was created that was assigned a value of 0 in the case of “Other hospital” for “type of hospital,” “0–199” for “bed capacity,” and “0–4 years” for “years of experience after obtaining CN or CNS qualification.” SPSS Statistics 21 software (IBM-SPSS, Inc., Chicago, IL, USA) was used for all analyses. Two-tailed tests were performed with an alpha level of .05. Because of the exploratory nature of the study, no correction for multiple testing was applied.

## Results

### Participant characteristics

The participants were 200 CNSs and 1,472 CNs with a mean length of clinical experience of 19.8 years. Job satisfaction was present in 38.45% of CNs and 49.00% of CNSs. In terms of workplace, 71.50% of the participants worked at designated cancer centers or hospitals, and the remaining ones worked at other hospitals (e.g., general hospitals or clinics) also involved in cancer care. Table 2 details the number of nurses working in different types of facilities or having a position title.

**Table 2:** Demographic characteristics.

			CNS		CN	
			*N*	%	*N*	%
Job satisfaction		Presence Absence	98 102	49.00 51.00	566 906	38.45 61.55
Clinical nursing experience (years)		5–9	25	12.50	93	6.30
		10–15	48	24.00	354	24.00
		16–20	71	35.50	402	27.30
		21–25	32	16.00	353	24.00
		26–30	20	10.00	194	13.20
		31–35	4	2.00	66	4.50
		36–35	0	0.00	7	0.50
		36–40	0	0.00	2	0.10
		41–45	0	0.00	1	0.10
Clinical experience as a CNS/CN (years)		Less than 5	148	74.00	895	60.80
		5 or more	52	26.00	577	39.20
Affiliation		Yes	82	41.00	409	27.80
Palliative care team		No	118	59.00	1064	72.20
	Cancer consultation and support center	Yes	54	27.00	83	5.60
		No	146	73.00	1390	94.40
	Chemotherapy room	Yes	26	27.00	402	27.30
		No	174	73.00	1071	72.70
	Radiotherapy room	Yes	4	2.00	65	4.40
		No	196	98.00	1408	95.60
	Outpatient department	Yes	28	14.00	356	24.20
		No	172	86.00	1117	75.80
	Ward department	Yes	59	29.50	629	42.70
		No	141	70.50	1404	57.30
Position		Manager	121	60.50	629	42.70
		Staff	79	39.50	843	57.30
Workplace		Cancer centers	175	87.50	1021	69.40
		Other hospitals	25	12.50	451	30.60
Bed capacity		Less than 200	9	4.50	165	11.20
		200 or more	191	95.50	1307	88.80

CNS: *n* = 200, CN: *n* = 1472

### Univariate analysis

The results of the chi-square test showed significant differences in the scores for 21 items for CNSs and 32 items for CNs (Tables 3 and 4). For CNSs, 11 differences were associated with activities, 7 with facilities, and 3 with the cancer care team. For CNs, 15 differences were associated with activities, 12 with facilities, and 5 with the cancer care team.

**Table 3:** Univariate analyses for CNSs.

			*N*	Satisfaction *n* (%)	Dissatisfaction *n* (%)	*P*
**Factors related to activity**						
Clinical nursing experience (years)		Less than 19	105	48 (45.70)	57 (54.30)	0.33
		19 or more	95	50 (52.60)	45 (47.40)	
Years of experience after acquiring CNS qualification (see Note)		Less than 5	148	62 (47.90)	86 (58.10)	0.00
		5 or more	52	36 (69.20)	16 (30.80)	
Presence in the same facility		Yes	115	46 (40.00)	69 (60.00)	0.16
• CNS		No	85	36 (42.40)	79 (57.60)	
• CN in palliative care		Yes	150	75 (50.00)	75 (50.00)	0.62
		No	50	23 (46.00)	27 (54.00)	
• CN in cancer pain management		Yes	114	55 (48.20)	59 (51.80)	0.81
nursing		No	86	43 (50.00)	43 (50.00)	
• CN in cancer		Yes	163	81 (49.70)	82 (50.30)	0.72
chemotherapy nursing		No	37	17 (45.90)	20 (54.10)	
• CN in radiation therapy		Yes	66	35 (53.00)	31 (47.00)	0.04
nursing		No	134	63 (47.00)	71 (53.00)	
• CN in breast		Yes	74	35 (47.30)	39 (52.70)	0.71
cancer nursing		No	126	63 (50.00)	63 (50.00)	
Affiliation		Yes	79	47 (59.50)	32 (40.50)	0.02
	• Palliative care team	No	121	51 (42.10)	70 (57.90)	
	• Cancer consultation and support center	Yes	54	35 (64.80)	19 (35.20)	0.01
		No	146	36 (24.70)	83 (75.30)	
	• Chemotherapy clinic	Yes	26	12 (46.20)	14 (53.80)	0.76
		No	174	86 (49.40)	88 (50.60)	
	• Radiotherapy room	Yes	4	3 (75.00)	1 (25.00)	0.29
		No	196	95 (48.50)	101 (51.50)	
	• Outpatient department	Yes	28	13 (46.40)	15 (53.60)	0.77
		No	172	85 (49.40)	87 (50.60)	
	• Ward department	Yes	59	13 (22.00)	46 (78.00)	0.00
		No	141	85 (60.30)	56 (39.70)	
Position		Manager	121	73 (60.30)	48 (39.70)	0.00
		Staff	79	25 (31.60)	54 (68.40)	
Role in educating others		Yes	185	96 (51.90)	89 (48.10)	0.00
		No	15	2 (13.30)	13 (86.70)	
Launched a department or patient group		Yes	79	26 (32.90)	53 (67.10)	0.00
		No	121	72 (59.50)	49 (40.50)	
Cross-departmental activities		Yes	151	93 (61.60)	58 (38.40)	0.00
		No	49	5 (10.20)	44 (89.80)	
Information exchanges or joint workshops etc. with other facilities		Yes	169	89 (52.70)	80 (47.30)	0.02
		No	31	9 (29.00)	22 (71.00)	
High ratings from senior staff		Yes	150	95 (63.30)	55 (36.70)	0.00
		No	50	3 (6.00)	47 (94.00)	
Appropriate staff allocation		Yes	86	53 (61.63)	33 (38.37)	0.02
		No	114	45 (39.47)	69 (60.53)	
**Factors related to facilities**						
Type of hospital		Cancer centers	175	88 (50.30)	87 (49.70)	0.34
		Other hospitals	25	10 (40.00)	15 (60.00)	
Bed capacity		Less than 200	9	3 (33.30)	6 (66.70)	0.40
		200 or more	191	95 (49.70)	96 (50.30)	
Advertisements for CNSs and CNs		Yes	185	91 (49.20)	71 (50.80)	0.85
		No	15	7 (46.70)	8 (53.30)	
Service system in four departments		Yes	152	81 (53.30)	71 (46.70)	0.03
		No	48	17 (35.40)	31 (64.60)	
Additional medical services Palliative care practice		Yes	111	58 (52.30)	53 (47.70)	0.30
		No	89	40 (44.90)	49 (55.10)	
• Cancer patient counseling		Yes	139	75 (54.00)	64 (46.00)	0.45
		No	61	23 (37.70)	38 (62.30)	
• Outpatient palliative care management		Yes	74	42 (56.80)	32 (43.20)	0.09
		No	126	56 (44.40)	70 (55.60)	
• Cancer pain palliation instructions		Yes	140	80 (57.10)	60 (42.90)	0.00
		No	60	18 (30.00)	42 (70.00)	
Requirements • Second opinion		Received	186	94 (53.40)	82 (46.60)	0.11
		Not received	14	4 (28.60)	10 (71.40)	
• Collaboration team and the primary care physician		Received	99	59 (59.60)	40 (40.40)	0.00
		Not received	101	39 (38.60)	62 (61.40)	
• Operation of cancer regional alliances path		Received	187	96 (51.30)	91 (48.70)	0.00
		Not received	13	2 (15.40)	11 (84.60)	
• Training of local healthcare professionals		Received	171	86 (50.30)	85 (49.70)	0.01
		Not received	29	12 (41.40)	17 (58.60)	
• Hospital cancer registry		Received	167	86 (51.50)	81 (48.50)	0.11
		Not received	33	12 (36.40)	21 (63.60)	
Education or support in obtaining CNS qualification of the assigned facilities		Yes	99	70 (70.70)	29 (29.30)	0.00
		No	101	28 (27.70)	73 (72.30)	
Education or support in obtaining CN qualification of the assigned facilities		Yes	156	92 (59.00)	64 (41.00)	0.00
		No	44	6 (13.60)	38 (86.40)	
**Factors related to the cancer care team**						
Coordination among healthcare professionals		Yes	194	98 (50.50)	96 (49.50)	0.00
		No	6	0 (0.00)	6 (100.00)	
Independent activities		Yes	98	96 (98.00)	2 (2.00)	0.00
		No	102	62 (60.80)	40 (39.20)	
Availability of conferences		Yes	187	96 (51.30)	91 (48.70)	0.01
		No	13	2 (15.40)	11 (84.60)	
Job type• Pharmacist		Involvement	183	90 (49.20)	93 (50.80)	0.44
		Non-involvement	17	8 (47.10)	9 (52.90)	
	• Medical social worker	Involvement	175	86 (49.10)	89 (50.90)	0.92
		Non-involvement	25	12 (48.00)	13 (52.00)	
	• Nutritionist	Involvement	140	66 (47.10)	74 (52.90)	0.42
		Non-involvement	60	32 (53.30)	28 (46.70)	
	• Physical therapist	Involvement	132	61 (46.20)	71 (53.80)	0.27
		Non-involvement	68	37 (54.40)	31 (45.60)	
	• Occupational therapist	Involvement	85	38 (44.70)	47 (55.30)	0.30
		Non-involvement	115	60 (52.20)	55 (47.80)	
	• Speech therapist	Involvement	54	21 (38.90)	33 (61.10)	0.08
		Non-involvement	146	77 (52.70)	69 (47.30)	
	• Clinical psychologist	Involvement	103	55 (53.40)	48 (46.60)	0.20
		Non-involvement	97	43 (44.30)	54 (55.70)	

The threshold for years of experience was set at 5 years, because according to [32], 44.00% of CNs are hesitant to renew their 5-year contracts the first time. Reasons given for their hesitation include: “There is no satisfaction in the work” and they have “not been able to fully refresh.”

**Table 4:** Univariate analyses for CNs.

			*N*	Satisfied *n* (%)	Not satisfied *n* (%)	*P*
**Factors related to activity**						
Clinical nursing experience (years)		Less than 19	849	320 (37.70)	529 (62.30)	0.48
		19 or more	623	246 (39.50)	377 (60.50)	
Years of experience after acquiring CN qualification (see Note)		Less than 5	895	270 (30.20)	625 (69.80)	0.00
		5 or more	577	296 (51.30)	281 (48.70)	
Presence in the same facility• CNS		Yes	467	199 (42.60)	268 (57.40)	0.02
		No	1005	367 (36.50)	638 (63.50)	
	• CN in palliative care	Yes	962	390 (40.50)	572 (59.50)	0.02
		No	510	176 (34.50)	334 (65.50)	
	• CN in cancer pain management nursing	Yes	603	233 (38.60)	370 (61.40)	0.74
		No	869	333 (38.30)	536 (61.70)	
	• CN in cancer chemotherapy nursing	Yes	911	374 (41.10)	537 (58.90)	0.04
		No	561	192 (34.20)	369 (65.80)	
	• CN in radiation therapy nursing	Yes	310	137 (44.20)	173 (55.80)	0.01
		No	1162	429 (37.00)	733 (63.00)	
	• CN in breast cancer nursing	Yes	363	158 (43.50)	205 (56.50)	0.03
		No	1109	408 (36.80)	701 (63.20)	
Affiliation		Yes	409	186 (45.50)	223 (54.50)	0.00
• Palliative care team		No	1063	380 (35.70)	683 (64.30)	
	• Cancer consultation and support center	Yes	83	45 (54.20)	38 (45.80)	0.00
		No	1389	521 (37.50)	868 (62.50)	
	• Chemotherapy room	Yes	402	167 (41.50)	235 (58.50)	0.14
		No	1070	399 (37.30)	671 (62.70)	
	• Radiotherapy room	Yes	65	19 (29.30)	46 (70.70)	0.12
		No	1407	547 (38.90)	860 (61.10)	
	• Outpatient department	Yes	356	130 (36.50)	226 (63.50)	0.39
		No	1116	436 (39.10)	680 (60.90)	
	• Ward department	Yes	629	177 (28.10)	452 (71.90)	0.00
		No	843	389 (46.10)	454 (53.90)	
Position		Manager	629	177 (28.10)	452 (71.90)	0.00
		Staff	843	389 (46.10)	454 (53.90)	
Role in teaching other nurses		Yes	1359	533 (39.20)	826 (60.80)	0.00
		No	113	33 (29.20)	80 (70.80)	
Launched a department or patient group		Yes	877	305 (34.80)	572 (65.20)	0.00
		No	595	261 (43.90)	334 (56.10)	
Cross-departmental activities		Yes	1053	500 (47.50)	553 (52.50)	0.00
		No	419	66 (15.80)	353 (84.20)	
Information exchanges or joint workshops etc. with other facilities		Yes	1209	505 (41.80)	704 (58.20)	0.00
		No	263	61 (23.20)	202 (76.80)	
High ratings from senior staff		Yes	980	511 (52.10)	469 (47.90)	0.00
		No	492	55 (11.20)	437 (88.80)	
**Factors related to facilities**						
Type of hospital		Cancer centers	1021	412 (40.40)	609 (59.60)	0.02
		Other hospitals	451	154 (34.10)	297 (65.90)	
Bed capacity		Less than 200	165	75 (45.50)	90 (54.50)	0.05
		200 or more	1307	491 (37.60)	816 (62.40)	
Advertisements for CNSs and CNs		Yes	1273	501 (39.40)	772 (60.60)	0.07
		No	199	65 (32.70)	134 (67.30)	
Service system in the following four departments		Yes	883	361 (40.90)	522 (59.10)	0.02
		No	589	205 (34.80)	384 (65.20)	
Additional medical services• Palliative care practice		Yes	593	236 (39.80)	357 (60.20)	0.38
		No	879	330 (37.50)	579 (62.50)	
	• Cancer patient counseling	Yes	942	394 (41.80)	548 (58.20)	0.00
No		530	172 (32.50)	358 (67.50)		
	• Outpatient palliative care management	Yes	340	139 (40.90)	201 (59.10)	0.29
No		1132	427 (37.70)	705 (62.30)		
	• Cancer pain palliation instruction	Yes	830	349 (42.00)	481 (58.00)	0.00
No		642	217 (33.80)	425 (66.20)		
Requirements • Second opinion		Received	1021	476 (46.60)	725 (53.40)	0.05
		Not received	271	90 (33.20)	181 (66.80)	
	• Collaboration team and the primary care physician	Received	605	278 (46.00)	327 (54.00)	0.00
Not received		867	288 (33.20)	579 (66.80)		
	• Operation of cancer regional alliances path	Received	670	291 (43.40)	379 (56.60)	0.00
Not received		802	275 (34.30)	527 (65.70)		
	• Training of local healthcare professionals	Received	996	424 (42.60)	572 (57.40)	0.00
Not received		476	142 (29.80)	334 (70.20)		
	• Hospital cancer registry	Received	973	404 (41.50)	569 (58.50)	0.00
Not received		499	162 (32.50)	337 (67.50)		
Positive evaluation of CNS development		Yes	531	286 (53.90)	245 (46.10)	0.00
		No	941	280 (29.80)	661 (70.20)	
Positive evaluation of CN development		Yes	898	451 (50.20)	447 (49.80)	0.00
		No	574	115 (27.00)	459 (73.00)	
Appropriate staff allocation		Yes	604	319 (52.81)	285 (47.19)	0.00
		No	868	247 (28.46)	621 (71.54)	
**Factors related to the cancer care team**						
Coordination among healthcare professionals		Yes	1384	548 (39.60)	836 (60.40)	0.00
		No	88	18 (20.50)	70 (79.50)	
Independent activities		Yes	1090	542 (49.70)	548 (50.30)	0.00
		No	382	24 (6.30)	358 (93.70)	
Availability of conferences		Yes	1235	515 (41.70)	720 (58.30)	0.00
		No	237	51 (21.50)	186 (78.50)	
Job Type• Pharmacist		Involvement	1350	528 (39.10)	822 (60.90)	0.44
		Non-involvement	122	38 (31.10)	84 (68.90)	
	• Medical social worker	Involvement	1137	461 (40.50)	676 (59.50)	0.00
		Non-involvement	335	105 (31.30)	230 (68.70)	
	• Nutritionist	Involvement	987	398 (40.30)	589 (59.70)	0.35
		Non-involvement	485	168 (34.60)	317 (65.40)	
	• Physical therapist	Involvement	831	325 (39.10)	506 (60.90)	0.55
		Non-involvement	641	241 (37.60)	400 (62.40)	
	• Occupational therapist	Involvement	547	217 (39.70)	330 (60.30)	0.46
		Non-involvement	925	349 (37.70)	576 (62.30)	
	• Speech therapist	Involvement	307	119 (38.80)	188 (61.20)	0.90
		Non-involvement	1165	447 (38.40)	718 (61.60)	
	• Clinical psychologist	Involvement	537	233 (43.40)	304 (56.60)	0.00
		Non-involvement	935	333 (35.60)	602 (64.40)	

The threshold for years of experience was set at 5 years, because according to [32], 44.00% of CNs are hesitant to renew their initial 5-year contracts. Reasons given for their hesitation Include: “There is no satisfaction in the work” and they have “not been able to fully refresh.”

### Multivariable analysis

The results of the chi-square test were used in a logistic regression analysis to identify variables particularly important to the job satisfaction of CNSs and CNs. Table 5 presents the results of this analysis.

**Table 5:** Logistic regression analysis.

			OR	95% CI			*p*
CNS							
Factors related to							
	Activities	Belongs to palliative care team	2.64	1.08	–	6.45	0.04
		Presence of cross-departmental activities	7.06	1.95	–	25.54	0.00
		Positive evaluation from senior staff	13.15	3.19	–	54.19	0.00
		Presence of CN in radiation therapy nursing in the same institution	2.91	1.08	–	7.84	0.03
	Facilities	Positive evaluation of CNS development	7.35	2.96	–	18.29	0.00
		Medical service fees: Cancer pain palliation instruction charges	3.78	1.45	–	9.85	0.01
Team	Opportunity for independent activities	11.34	2.04	–	62.99	0.01
CN							
Factors related to							
	Activities	Belongs to ward department	0.49	0.38	–	0.66	0.00
		Opportunities for cross-departmental activities	2.24	1.57	–	3.19	0.00
		Positive evaluation from senior staff	4.88	3.46	–	6.46	0.00
		Appropriate staff allocation	1.75	1.35	–	2.27	0.00
		CN experience of more than 5 years	1.91	1.44	–	2.54	0.00
	Facilities	Bed capacity	0.33	0.20	–	0.54	0.04
		Positive evaluation of CNS development	1.37	1.01	–	1.86	0.04
		Positive evaluation of CN development	2.13	1.55	–	2.93	0.00
Team	Opportunity for independent activities	6.83	4.28	–	10.91	0.00

Adjusted for years of experience after acquiring a nursing license. Only variables that remained significant in the regression models are shown in this table.

#### Multivariable analysis of job satisfaction in CNSs

In terms of the factors related to activities, job satisfaction was present when the participant belonged to the palliative care team [odds ratio (OR) = 2.64], cross-departmental activities could be performed (OR = 7.06), the participant received a favorable rating from senior staff (OR = 13.15), and a CN was involved in radiation therapy nursing in the same institution (OR = 2.91). Of the factors related to facilities, job satisfaction was found when there was a high rating for CNS development in the institution (OR = 7.35) and the participant belonged to an institution where an additional pain relief management fee was charged (OR = 3.78). Of the factors related to the cancer care team, job satisfaction was found when independent activities could be performed (OR = 11.3) (Cox-Snell *R*^2^ = 0.49; Nagelkerke *R*^2^ = 0.65).

#### Multivariable analysis of job satisfaction in CNs

Of the factors related to activities, working on a ward was associated with the absence of job satisfaction (OR = 0.49). In contrast, job satisfaction was present when cross-departmental activities could be performed (OR = 2.24), a high rating was received from senior staff (OR = 4.88), there was appropriate staff allocation in each ward or department (OR = 1.75), and the participant had at least five years of experience after acquiring a CN qualification (OR = 1.91). Of the factors related to facilities, job satisfaction was present when the capacity was less than 200 beds (OR = 0.33) and there was a high rating of CNS and CN development in the institution (OR = 1.37 and 2.13, respectively). Of the factors related to the cancer team, job satisfaction was present when independent activities could be performed (OR = 6.83) (Cox-Snell *R*^2^ = 0.33; Nagelkerke *R*^2^ = 0.45).

## Discussion

In this study on CNs and CNSs working in cancer care in Japan, we identified numerous factors related to activities, facilities, or the cancer team that influenced job satisfaction.

Of the factors related to activities, opportunities for cross-departmental activities and positive evaluation from senior stuff were common to CNs and CNSs.

**Fig 1:**
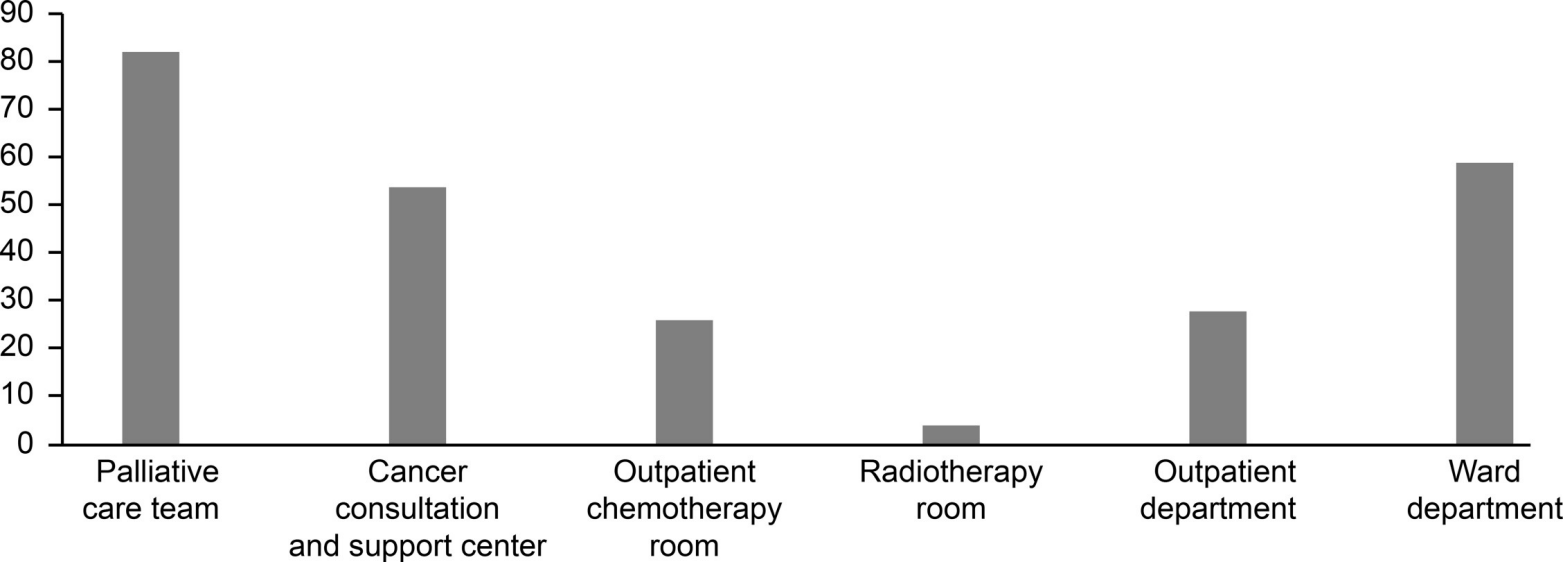
Total number of CNSs in each affiliation.

In our study, CNSs were often affiliated with departments engaged in cross-departmental activities throughout the entire facility (Fig 1).

**Fig 2:**
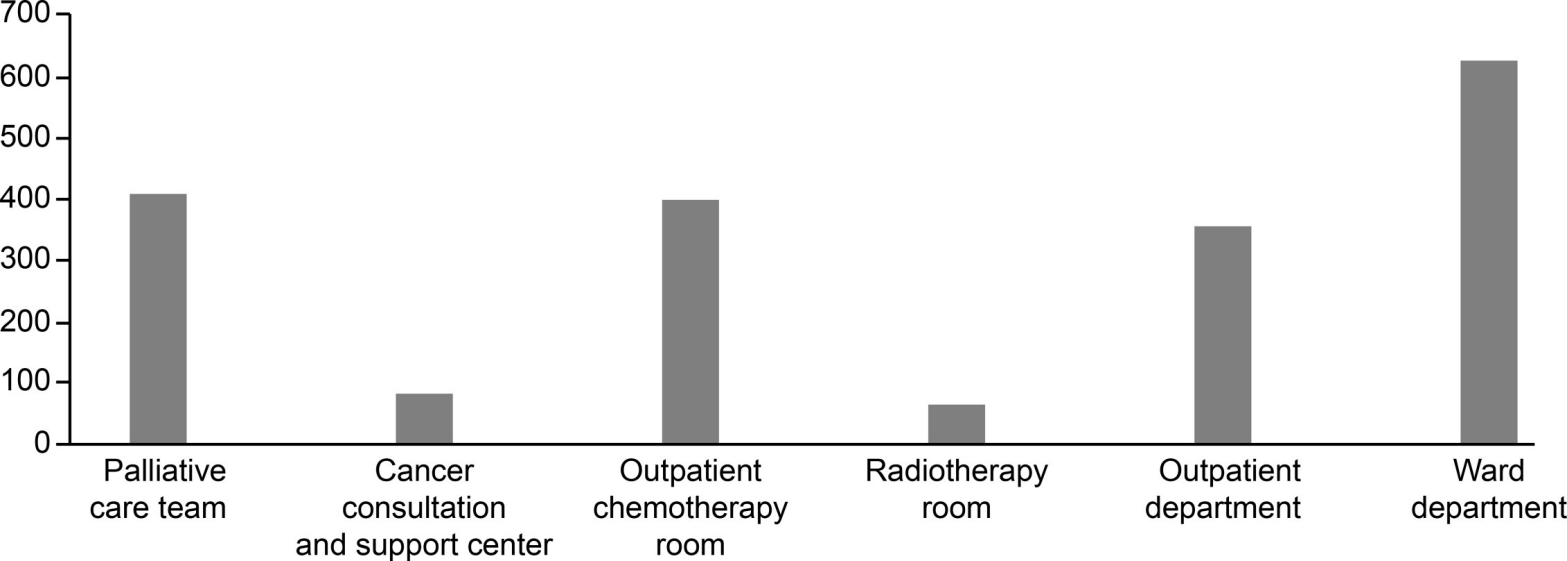
Total number of CNs in each affiliation.

Cross-departmental activities increased the job satisfaction of CNSs and CNs. However, these mainly involved activities by the palliative care team and the counseling and support center. In this study, only 409 members of palliative care teams and 83 members of the counseling and support centers were surveyed, and a high number of respondents (1,404) were affiliated with a single department (Fig 2). In facilities without CNSs, consultations in other wards and care for patients during radiotherapy require specialized abilities and offer CNs an opportunity to demonstrate their professional skills, which is thought to increase job satisfaction [33, 34].

Our findings are consistent with those of a previous study that showed that consciously conducting professional activities as a specialist increases work engagement, which influences job satisfaction [35]. Furthermore, the feeling that a job is worthwhile and that one’s performance in the role has been noticed have been correlated with increased job satisfaction [36]. Affective commitment is associated with increased job satisfaction and attachment to the organization [37, 38]. The results here suggest that it would be beneficial to provide CNSs and CNs with opportunities to maximize their abilities, which would enhance their affective commitment and job satisfaction.

CNs reported the presence of job satisfaction when staffing was appropriate in the department and when opportunities were available to perform cross-departmental and independent activities. However, job satisfaction was lower among CNs working on a ward, because doing so is associated with a higher level of exhaustion, which reduces job efficacy. Consistent with our findings, the appropriate staff allocation in wards or departments has been associated with a higher level of job satisfaction among CNs [17]. Therefore, flexible staffing is considered a guarantee that CNs will complete their activities. Job satisfaction increased at least five years after obtaining a CN qualification and when the individual received good evaluations from superiors. In addition, length of experience has been cited as a factor influencing the career development of CNs, and specialty careers with greater autonomy are associated with higher job satisfaction [39, 40]. For CNs, job satisfaction increased after the fifth year, when they can be fully active and solve problems independently.

In addition, job satisfaction was higher when the development of CNSs and CNs was conducted more proactively. Those who received positive evaluations from their senior staff tended to report the presence of job satisfaction. Because work engagement is increased by the availability of resources such as support from senior staff and colleagues [41], and lack of social support from senior staff is associated with the intention to quit a job [42], a high rating and support from senior staff is thought to increase the job satisfaction of CNSs and CNs. Thus, for CNSs and CNs to develop their professional abilities [43], receiving a favorable evaluation from a supervisor is essential.

Although previous studies found the presence of job satisfaction among CNSs and CNs who are more active in teaching, the absence of supportive colleagues reportedly influence job satisfaction as well [41–43]. Moreover, affective commitment, which involves equating one’s personal values with those of the organization, is strongly associated with job motivation in nurses [44] and enhances job satisfaction. Importantly, the perception of support from colleagues is related to this affective commitment [45, 46]. For example, perceptions of mutual support between CNSs and CNs seemed to enhance job satisfaction.

Of the factors related to facilities, a positive evaluation of the development of CNs and CNSs was associated with job satisfaction in both CNs and CNSs.

CNSs’ job satisfaction was significantly higher when institutions charged additional fees for pain relief management. The fee for opioid use in Japan is approximately 5.00% of that in Western countries [47]. However, it is difficult to determine whether appropriate pain care is provided in Japan. In this context, there is often misunderstanding due to the lack of knowledge about opioids of both patients and healthcare professionals [48]. Therefore, the reevaluated and newly established system for medical service fees that provides instructions on pain palliation in cancer treatment may offer a sense of fulfillment and satisfaction to CNSs, because they are able to provide care that alleviates pain and improves their patients’ quality of life.

Moreover, job satisfaction was higher among CNs who worked at an institution with a capacity of less than 200 beds. Reportedly, professional autonomy is affected by the size and management system of the organization, not by individual traits [40]. The opinions of nurses are addressed by administrators more readily in smaller institutions than in larger ones, and they can demonstrate their competency more easily, which may explain the association between bed capacity and job satisfaction. Of the factors related to the cancer care team, opportunity for independent activities was associated with the job satisfaction of both CNs and CNSs. To experience job satisfaction, individuals with an internal locus of control need an environment in which they can effectively use their roles to determine the outcomes of their actions [35]. Hence, for CNSs and CNs to provide care as intended, an environment is required in which they can fully use their cultivated knowledge, skills, and designated roles. This tendency toward an internal locus of control might have increased job satisfaction in the current study.

Certified nursing was an outcome of the notion that nurses should collaborate and engage in cross-sectional activities beyond their wards. Engaging in such activities likely increases collaboration and the job satisfaction of CNSs. It is important that organizations adopt practices based on the findings of this study. Moreover, the working conditions and job satisfaction of CNSs and CNs reported in this study are closely connected with policy. Therefore, our findings should be used to guide policy in Japan in the future.

Several previous studies have been conducted on the job satisfaction of nurses in Japanese institutions. They highlight the complex nature of factors influencing job satisfaction and importance thereof in staff retention [49–55].

Our study has several limitations. Because fewer responses were obtained from CNSs (only 200 of the total 483 CNSs) than CNs (1,472 responses), the 95% confidence interval of the logistic regression was rather large. In addition, the effects of age and sex were not considered. The group sizes of participants working in radiotherapy or wards with lower bed capacity were skewed. Finally, because of the explorative character of our study, we did not correct for multiple testing. Therefore, these results must be generalized with caution.

## Conclusion

Our study identified the factors related to activities, facilities, and the cancer care team associated with the job satisfaction of CNSs and CNs, and highlighted potential avenues through which to enhance job satisfaction through the Basic Plan to Promote Cancer Control in Japan. Whereas the majority of the factors identified were shared by CNs and CNSs, several were unique to one of the groups. Moreover, the findings have implications for hospital administrators aiming to retain staff who might otherwise be hesitant to stay because of job dissatisfaction. Finally, changes regarding the CN certification may be beneficial.

## Supporting information

S1 Data (XLSX)

S2 Data (XLSX)
