# Supplementary material for: Factors associated with the job satisfaction of certified nurses and nurse specialists in cancer care in Japan: Analysis based on the Basic Plan to Promote Cancer Control Programs
Source: PLoS One. 2020 May 18;15(5):e0232336. doi: 10.1371/journal.pone.0232336 (PMC7233545; doi:10.1371/journal.pone.0232336)
Supplement: Attachment — Submitted filename: Response to Reviewers.docx [file pone.0232336.s003.docx]

**Response to the Editor and Reviewers

Editor:**

When submitting your revision, we need you to address these additional requirements. Please ensure that your manuscript meets PLOS ONE's style requirements, including those for file naming.

**Response:** We have renamed the files in accordance with the journal requirements and rechecked the text for compliance with the formatting requirements.

We note that you have stated that you will provide repository information for your data at acceptance. Should your manuscript be accepted for publication, we will hold it until you provide the relevant accession numbers or DOIs necessary to access your data. If you wish to make changes to your Data Availability statement, please describe these changes in your cover letter and we will update your Data Availability statement to reflect the information you provide.

**Response:** We confirm that we will make the manuscript data publicly available upon acceptance of the article and agree with the outlined procedure.

Please amend your current ethics statement to include the full name of the ethics committee/institutional review board(s) that approved your specific study. Once you have amended this/these statement(s) in the Methods section of the manuscript, please add the same text to the “Ethics Statement” field of the submission form (via “Edit Submission”). For additional information about PLOS ONE ethical requirements for human subjects research, please refer to <http://journals.plos.org/plosone/s/submission-guidelines#loc-human-subjects-research>.

**Response:** We have specified the name of the Ethics Committee (“Institutional Ethics Committee of the Kyoto University Graduate School of Medicine and Faculty of Medicine Hospital”) in the Methods section and the ethical statement.

PLOS requires an ORCID iD for the corresponding author in Editorial Manager on papers submitted after December 6th, 2016. Please ensure that you have an ORCID iD and that it is validated in Editorial Manager. To do this, go to ‘Update my Information’ (in the upper left-hand corner of the main menu), and click on the Fetch/Validate link next to the ORCID field. This will take you to the ORCID site and allow you to create a new iD or authenticate a pre-existing iD in Editorial Manager. Please see the following video for instructions on linking an ORCID iD to your Editorial Manager account: <https://www.youtube.com/watch?v=_xcclfuvtxQ>.

**Response:** We have provided the ORCiD ID for the corresponding author as requested.

**Reviewer #1:**

The manuscript touches an important topic by trying to assess Job satisfaction of certified nurse specialists and certified nurses in cancer care centers in Japan. However, I have some doubts how this study was conducted:
Title:
- Your title of this study is “to assess Job satisfaction of certified nurse specialists and certified nurses in cancer care centers in Japan”. However; there is no finding in your study which shows the level of job satisfaction among these two group of nurses. Therefore, you have to either present this finding or modify your title.

**Response:** We thank the reviewer for this helpful comment. Job satisfaction was present in 38.45% of CNs and 49% of CNSs, but absent in 61.55% of CNs and 51% of CNSs. We have added this information to the revised manuscript (page 12, lines 202-203 and Table 2). Furthermore, to more exactly convey the topic and findings of the paper, we have modified the title to: “Factors affecting the job satisfaction of certified nurse specialists and certified nurses in cancer care in Japan: Analysis based on the Basic Plan to Promote Cancer Control Programs.”

Abstract:
- In the background part of abstract, information about nurses’ job satisfaction such as level of job satisfaction and burden of job dissatisfaction on patients should be presented from the available literature.

**Response:** We have added a statement on the level of job satisfaction and its consequences to the background part of the abstract. Due to the 300 words limit for the abstract, we had to keep the statement concise (page 2, lines 21-23).

-I am not clear with your sample size calculation and sampling procedure. How 200 certified nurse specialist and 1,472 certified nurses were recruited and participated in the study?

**Response:** The sample size was restricted by the number of eligible qualified and recruited CNs and CNSs. Overall, 2,378 eligible CNs were identified and contacted; 1,486 replied, and 1,472 among them provided valid answers. Overall, 514 eligible CNSs were identified and contacted; 210 replied, and 200 among them provided valid answers.

-The authors should indicate the level of job satisfaction of both certified nurse specialist and certified nurses

**Response:** Job satisfaction was present in 38.45% of CNs and 49% of CNSs but was absent in 61.55% of CNs and 51% of CNSs. We have added this information to the manuscript (page 12, lines 202-203 and Table 2).

-Indicate all variables associated with job satisfaction separately for certified nurse specialist and certified nurses, and mention their respective AOR with CI.

**Response:** All variables associated with job satisfaction were listed separately, and their odds ratios and p-values were specified. Unfortunately, the word limit for the abstract (300 words) did not allow listing also the 95% CI in the abstract; however, this information is described in the main text.

-The authors should present the conclusion of their finding in the conclusion section of the abstract

**Response:** We have stated the conclusion of the study in the conclusion section of the abstract (page 3, lines 45-48).

Introduction:
- Based on the available literature, the authors should present the level of job satisfaction among nurses in global, regional and local perspectives

**Response:** The introduction has been modified (page 5, lines 86-91).

- The authors should also show what the literature says about the burden/impact of nurses’ job dissatisfaction on cancer patients. The authors should also mention important factors influencing job satisfaction based on the available literature on this particular topic

**Response:** We have mentioned important factors affecting job satisfaction in the introduction (page 5, lines 86-91). Unfortunately, the effect of nurse job dissatisfaction on cancer patient outcomes has not been investigated well. We have added that as a future research direction in the discussion.

- I recommend the authors to remove table 1 from introductory part of this manuscript, because information in the table are already mentioned in narration

**Response:** We have removed Table 1 from the revised version of the manuscript.

- The statement “No study in the study area” don’t justify the need of your study. The authors should show gap of previous studies (global perspectives) and justify the need of your studies

**Response:** We have modified the justification for the study (page 6, lines 98-102).

Methods:
- I am not sure whether the level job satisfaction of nurses was measured in this study. If so, which tool was used to measure this job satisfaction? Was this tools validity and reliability checked?

**Response:** To measure job satisfaction, we asked the question, "Are you satisfied with your job?” Originally, we used a scale with three levels, “satisfied,” “somewhat satisfied,” and “dissatisfied.” However, upon review, we combined the first two levels to yield a two-level scale, “satisfied” and ”dissatisfied.” We have added this information to the text of the manuscript (page 8, lines 142-145).

- Why you conducted pre-test on only 4 nurses? As a rule, on how much percentage of sample size pre-test should be conducted?

**Response:** Only 4 nurses were included in the pre-test due to organizational and technical reasons despite our intention to conduct a more extensive pre-test. Since the pre-test was conducted in person and was time-consuming, it was difficult to recruit nurses willing to commit the time for the pre-test. Furthermore, the nurses we managed to recruit were at a geographical distance from us, which necessitated travel to their institutions, further increasing the time load.

- How you determined the sample size? What was your sampling procedure? The authors should describe the mechanism how participants were recruited and participated in the study

**Response:** As mentioned earlier in our response, we contacted all eligible qualified CNs and CNSs. Overall, there were 2,378 eligible CNs; 1,486 replied, and 1,472 among them provided valid answers. Overall, there were also 514 eligible CNSs; 210 replied, and 200 among them provided valid answers.

- Since the data were collected through mail, I doubt how the informed consent was obtained.

**Response:** The contacted CNs and CNSs had the opportunity to select also the item “I do not agree to participate.” in the questionnaire we sent them.

- My major issue with this research is how job satisfaction was assessed/measured and categorized as “satisfaction” and “dissatisfaction”. Therefore, the authors should clearly explain this in the methods

**Response:** To measure job satisfaction, we asked the question, "Are you satisfied with your job?” Originally, we used a scale with three levels, “satisfied,” “somewhat satisfied,” and “dissatisfied.” However, upon review, we combined the first two levels to yield a two-level scale, “satisfied” and ”dissatisfied.”

- Your method of analysis is not clear. Please, explain your method of analysis in detail

**Response:** The description of the data analysis has been revised in the text.

Results:
- Your objective is to assess Job satisfaction of certified nurse specialists and certified nurses in cancer care centers in Japan. Where is the result which indicate level of job satisfaction for certified nurse specialist and certified nurses? What percentages of certified nurse specialists and certified nurses were satisfied and dissatisfied with their job?

**Response:** Job satisfaction was present in 38.45% of CNs and 49% of CNSs, and absent in 61.55% of CNs and 51% of CNSs (page 12, lines 202-203 and Table 2).

Discussion:
- The authors should re-write the discussion. The authors only stated and discussed their finding and didn’t compare their finding with other study findings. The authors should discuss only the key finding of their study with previous study findings.

**Response:** We have modified the discussion to focus more on the comparison of our findings with the results of previous investigations.

Conclusion:
- The authors should write the conclusion of the study finding separately

**Response:** The Conclusion has been separated into an individual section (page 27, lines 390-396).

**Reviewer #2:** Thank you for the opportunity to review this paper. This paper is novel in that it is the first to evaluate the factors associated with job satisfaction among nurses involved in cancer care in Japan, which is an important group of healthcare professionals.

Introduction
1) In Table 1, years of clinical experience to qualify for CNSs for university graduates is stated as “0” years. Please confirm this is correct.

**Response:** We thank the Reviewer for this comment. The second Reviewer of the manuscript requested that we remove Table 1 due to the presentation of the data in the text. Therefore, Table 1 has been removed from the revised version of the manuscript.

2) Reference 22 appears to be out of context w.r.t addressing job satisfaction among nurses. Please confirm if the reference is appropriate. (line 91)

**Response:** We thank the Reviewer for pointing out this inconsistency. We have removed reference 22 from the revised manuscript.

Methods
1) The interviews conducted (line 111) needs to be further clarified. E.g., with who?

**Response:** We meant the pre-test interviews with CNs. To avoid confusion, this phrase was removed from the revised version of the manuscript.

2) “a logistic regression analysis was conducted with satisfaction or dissatisfaction as the dependent variable” (line 183)- please provide more information to explain how the dependent variable is being determined. Given the proposed title of the manuscript, it is also important to report on job satisfaction status as descriptive statistics in Table 3.

**Response:** We have reported on job satisfaction in the descriptive statistics Table, which is currently Table 2 in the revised manuscript. To measure job satisfaction, we asked the question, "Are you satisfied with your job?” Originally, we used a scale with three levels, “satisfied,” “somewhat satisfied,” and “dissatisfied.” However, upon review, we combined the first two levels to yield a two-level scale, “satisfied” and ”dissatisfied.” (page 12, lines 202-203 and Table 2).

3) “years of experience as the control variable” (line 185) refers to years of experience after acquiring nursing license or after acquiring CN qualification?

**Response:** "Years of experience after acquiring nursing license" was used as a control variable, whereas "years of experience after acquiring CNS or CN qualification" was utilized as an independent variable. We have clarified this information in the text.

Results:
Overall: The regression analysis for CNSs (n=200) is under-powered given the large no. of independent variables involved (which i suppose to be 20 of them)

**Response:** We contacted all eligible qualified CNs and CNSs. Overall, there were 514 eligible CNSs; 210 replied, and 200 among them provided valid answers. Thus, the CNS sample size was limited by the number of individuals eligible and willing to participate. We have stated this fact as a limitation of our study.

1) “remaining participants worked at other hospitals (e.g., general hospitals or clinics)” (line 196-197). It is unclear if these participants were also involved in cancer care? This appears to contradicts the statement under section-Participants which stated that “A total of 1,696 nurses working in cancer centers and hospitals responded to the mailed questionnaire. (line 160-161)”. Please clarify.

**Response:** We apologize for this unclarity. These participants worked in general hospitals or clinics, but they were also involved in cancer care. We have modified both statements, to which the Reviewer referred, to clarify this point (page 12, lines 204-206).

2) “51.6% of the nurses had a position title (e.g., head or chief nurse), whereas the others were staff nurses” (line 203), is this established based on “position” in Table 3 where managers are “head or chief nurse”? If so, the reported % does not seem to be correct.

**Response:** We apologize for this inconsistency and have changed the value to the correct value of 60.5%.

3) I noticed significant differences in 5 (not 3) items with cancer team care for Table 5. Please correct text under results accordingly (line 208 and line 211).

**Response:** We thank the Reviewer for pointing out this inconsistency; we have corrected it (page 14, lines 219-220).

4) “The results of the χ2 test were used in a logistic regression analysis to identify variables particularly important to job satisfaction among CNSs and CNs.” (line 228-229). This needs to be highlighted in the methodology and it is unclear how results from chi square tests were used to identify variables for inclusion in logistic regressions? Was it that only items with p<0.05 at univariate analysis were selected as explanatory variables in the logistic regressions? If so, why is “presence of CN in radiation therapy nursing” included and remained significant in the regression analysis for CNS (Table 6) when it was reported as “n.s” in the univariate analysis for CNS. Please clarify. I have concern on the over-loading of the model, particularly given the small sample in CNS analysis. Is correction for multiple testing considered?

**Response:** We thank the Reviewer for pointing out this unclarity. We have specified in the revised manuscript that only items with *p* < 0.05 in the univariate analysis were selected as explanatory variables in the logistic regressions (page 11, lines 186-188). The presence of CN in radiation therapy nursing “n.s” report in the univariate analysis for CNS was a mistake that we have corrected. Due to the exploratory character of our study, we did not correct for multiple testing. We have stated this fact as a limitation in the revised version of the manuscript (page 27, lines 387-388).

5) Table 6 shows only variables that remain significant in regression models? If so, please state this as legend in the Table.

**Response:** We have stated in the note to the table in the revised manuscript that only variables that remain significant in regression models are shown in Table 6.

6) “when a CN was involved in radiation therapy nursing within the same institution” (line 242-243); please clarify if this is a factor related to activity or facilities? It is reported as a factor for facilities.

**Response:** We thank the Reviewer for pointing out this inconsistency and apologize for it. “When a CN was involved in radiation therapy nursing within the same institution” was a factor associated with facilities. We have corrected the text accordingly.

7) Suggest to use the terms “presence” or “absence” of job satisfaction instead of “higher” or “lower” level of job satisfaction when reporting the results.

**Response:** We have replaced the phrases “higher” or “lower” level of job satisfaction with “presence” or “absence” of job satisfaction.

Discussion
1) “a high number of respondents (1,404) were affiliated with a single ward” (line 294). Figure 2 shows that less than 700 (based on bar height) were involved in ward department. Please clarify.

**Response:** We thank the reviewer for pointing out this inconsistency. By “affiliated with a single ward” we meant “affiliated with a single department,” including a ward or outpatient department, as well as a chemotherapy or radiotherapy room. We have modified the phrase in the text correspondingly (page 23, lines 305-306).
