# Supplementary material for: Factors associated with the job satisfaction of certified nurses and nurse specialists in cancer care in Japan: Analysis based on the Basic Plan to Promote Cancer Control Programs
Source: PLoS One. 2020 May 18;15(5):e0232336. doi: 10.1371/journal.pone.0232336 (PMC7233545; doi:10.1371/journal.pone.0232336)
Supplement: Attachment — Submitted filename: Response to reviewers.docx [file pone.0232336.s004.docx]

**Response to the Editor and Reviewers

Academic Editor:**

Please consider the comments by the third reviewer pertaining to data analysis. You may wish to revise your analysis or justify your analytical approach in the light of these comments.

**Response:** Thank you for the opportunity to revise our manuscript. We have conducted additional statistical analysis in accordance with the comments of Reviewer 3.

**Reviewer #2:**

The authors have adequately addressed most of comments except one:

1) "when a CN was involved in radiation therapy nursing within the same institution” (line 248-252 in the clean version); please clarify if this is a factor related to activity or facilities? The author replied in his/her comment that this is a factor related to facilities which is confusing and it does not tally with information presented in the tables. I did not also see any change in the text.

**Response:** We apologize for this mistake. In the revised manuscript, we changed this to “when a CN was involved in radiation therapy nursing within the same institution” as a factor related to activity (page 21, lines 234-235).

Others:

1) Line 217 to 218. All reported numbers should be updated. 20->21, 30->32, 10->11

**Response:** Thank you for pointing out this inconsistency. We have updated the reported numbers accordingly (page 14, lines 205-208).

**Reviewer #3:**

The use of the abbreviations is not consistent. For example, CN or CNs, CNSs. I would suggest to remove all acronyms from the abstract in the least and standardize their use throughout the manuscript. The use of the acronyms has to be grammatically correct i.e. used in singular or plural forms. Consider using ‘certified nurse or nurse specialists’ in the sentences. Please spell chi-square in full.

**Response:** Thank you for this comment. We have ensured that the use of abbreviations in the revised version of the manuscript is consistent by introducing the following modifications: (1) We have removed all acronyms from the abstract. (2) In the main text, we have used CN and CNS in singular form and CNs and CNSs in plural form. (3) In the abstract, we have used the expression “certified nurses or nurse specialists” as suggested. (4) We have spelled out “chi-square” in full.

Since this is a cross-sectional study, refrain from using terms such as ‘predictive’. Change these to ‘association’ instead.

**Response:** We deleted all terms indicating a causative effect such as “predictive” and changed them to terms indicating an “association” as requested.

State all proportions with upto one decimal points and other estimates upto 2 or 3 (keep it consistent).

**Response:** We have modified the text to consistently present estimates up to 2 decimal points.

Please state full p values instead of asterisks in the tables.

**Response:** We have provided the full p-values instead of asterisks in the tables.

I am unable to understand the significant of having the analysis based on the Basic Plan to Promote Cancer Control Programs. Perhaps in the introduction this should be described and its relevance to this study should be highlighted. It may also be necessary to state why it was of interest to study CN and CNS groups separately. Just stating there were no previous studies may not be enough.

**Response:** In the introduction, we now elaborate why we chose to base the analysis on the Basic Plan to Promote Cancer Control Programs (page 5, lines 75-77 of the clean copy). The roles of certified nurses and nurse specialists are regulated by the Basic Plan to Promote Cancer Control Programs. Therefore, it is important to consider this plan when assessing nurses’ job satisfaction. Furthermore, considering the plan would help us make more relevant suggestions for measures from the Japanese Ministry of Health, Labour and Welfare to improve job satisfaction. The roles of certified nurses and nurse specialists partially overlap, but also differ in several aspects. Therefore, it is important to analyze the factors influencing job satisfaction in these groups separately to identify overlapping and distinct factors. This also facilitates the identification of measures that can improve the job satisfaction of certified nurses and nurse specialists.

The discussion is not informative and needs to be clearly focused on the main findings of the study and the distinctions between the two groups and their practice implications.

**Response:** We have extensively revised the discussion accordingly.

In terms of analysis, some of the groups are highly skewed eg. Proportions working in radiotherapy, wards with lower bed capacity. Years of clinical nurse experience also needs to be carefully looked at since this has been used as control variable in the logistic regression. Although authors justify using 5 year cut off, I believe this again would be very skewed population given the mean duration was 19 years in the sample. The univariate analysis need to relooked at carefully before presenting multivariable findings. On that note, the correct term would be ‘multivariable’ instead of ‘multivariate’

**Response:** Thank you for your comment. In the revised manuscript, we conducted an univariate analysis on the association of job experience of “less than 19 years”/“19 years and above” with “job satisfaction” (page 14, Table 3). No significant association was found for both certified nurses and nurse specialists. In addition, we replaced the term “multivariate” with “multivariable.” We have stated in the limitations statement that some groups such as participants working in radiotherapy or wards with lower bed capacity were skewed.

Overall, I found this article quite difficult to follow. Given that it is currently quite long, authors need to edit the content carefully after considering the above comments. This could be achieved by shortening the introduction, avoiding repetitions between the tables and text in the results and trimming the discussion.

**Response:** We edited the introduction for conciseness and shortened it. We also checked the text for repetition between the tables and text and removed any such cases. Finally, we modified the discussion and edited it for conciseness.

Parts of the manuscript also need better flow and focus. For example, why is the statement ‘The study adheres to the STROBE checklist’ included in the job satisfaction questionnaire section? The intent of mentioning ‘Basic Plan to Promote Cancer Control Programs’ is not clear. Analysis needs to be reviewed thoroughly and reanalyzed as necessary to be eligible for publication.

**Response:** We moved the statement regarding the STROBE checklist (page 10, lines 170-171). Furthermore, we clarified the intent of mentioning the Basic Plan to Promote Cancer Control Programs in the introduction (page 5, lines 75-77). We also reviewed the statistical analysis and conducted additional statistical analysis.

**Additional comment:**

The PLOS Data policy requires authors to make all data underlying the findings described in their manuscript fully available without restriction, with rare exception (please refer to the Data Availability Statement in the manuscript PDF file). The data should be provided as part of the manuscript or its supporting information, or deposited to a public repository. For example, in addition to summary statistics, the data points behind means, medians and variance measures should be available. If there are restrictions on publicly sharing data—e.g. participant privacy or use of data from a third party—those must be specified

Reviewer #2: Yes

Reviewer #3: No

**Response:** If the manuscript is accepted for publication, we will make the data fully available in a public repository.
